# Text-Messaging, Online Peer Support Group, and Coaching Strategies to Optimize the HIV Prevention Continuum for Youth: Protocol for a Randomized Controlled Trial

**DOI:** 10.2196/11165

**Published:** 2019-08-09

**Authors:** Dallas Swendeman, Elizabeth Mayfield Arnold, Danielle Harris, Jasmine Fournier, W Scott Comulada, Cathy Reback, Maryann Koussa, Manuel Ocasio, Sung-Jae Lee, Leslie Kozina, Maria Isabel Fernández, Mary Jane Rotheram, Adolescent Medicine Trials Network (ATN) CARES Team

**Affiliations:** 1 University of California, Los Angeles Department of Psychiatry & Biobehavioral Sciences Los Angeles, CA United States; 2 Department of Family and Community Medicine UT Southwestern Medical Center Dallas, TX United States; 3 Tulane University School of Medicine Department of Pediatrics New Orleans, LA United States; 4 Nova Southeastern University College of Osteopathic Medicine Fort Lauderdale, FL United States

**Keywords:** adolescents, HIV/AIDS, mHealth, homelessness, MSM, transgender, prevention

## Abstract

**Background:**

America’s increasing HIV epidemic among youth suggests the need to identify novel strategies to leverage services and settings where youth at high risk (YAHR) for HIV can be engaged in prevention. Scalable, efficacious, and cost-effective strategies are needed, which support youth during developmental transitions when risks arise. Evidence-based behavioral interventions (EBIs) have typically relied on time-limited, scripted, and manualized protocols that were often delivered with low fidelity and lacked evidence for effectiveness.

**Objective:**

This study aims to examine efficacy, implementation, and cost-effectiveness of easily mountable and adaptable, technology-based behavioral interventions in the context of an enhanced standard of care and study assessments that implement the guidelines of Centers for Disease Control and Prevention (CDC) for routine, repeat HIV, and sexually transmitted infection (STI) testing for high-risk youth.

**Methods:**

Youth aged between 12 and 24 years (n=1500) are being recruited from community-based organizations and clinics serving gay, bisexual, and transgender youth, homeless youth, and postincarcerated youth, with eligibility algorithms weighting African American and Latino youth to reflect disparities in HIV incidence. At baseline and 4-month intervals over 24 months (12 months for lower-risk youth), interviewers monitor uptake of HIV prevention continuum steps (linkage to health care, use of pre- or postexposure prophylaxis, condoms, and prevention services) and secondary outcomes of substance use, mental health, and housing security. Assessments include rapid diagnostic tests for HIV, STIs, drugs, and alcohol. The study is powered to detect modest intervention effects among gay or bisexual male and transgender youth with 70% retention.

Youth are randomized to 4 conditions: (1) enhanced standard of care of automated text-messaging and monitoring (AMM) and repeat HIV/STI testing assessment procedures (n=690); (2) online group peer support via private social media plus AMM (n=270); (3) coaching that is strengths-based, youth-centered, unscripted, based on common practice elements of EBI, available over 24 months, and delivered by near-peer paraprofessionals via text, phone, and in-person, plus AMM (n=270); and (4) online group peer support plus coaching and AMM (n=270).

**Results:**

The project was funded in September 2016 and enrollment began in May 2017. Enrollment will be completed between June and August 2019. Data analysis is currently underway, and the first results are expected to be submitted for publication in 2019.

**Conclusions:**

This hybrid implementation-effectiveness study examines alternative models for implementing the CDC guidelines for routine HIV/STI testing for YAHR of acquiring HIV and for delivering evidence-based behavioral intervention content in modular elements instead of scripted manuals and available over 24 months of follow-up, while also monitoring implementation, costs, and effectiveness. The greatest impacts are expected for coaching, whereas online group peer support is expected to have lower impact but may be more cost-effective.

**Trial Registration:**

ClinicalTrials.gov NCT03134833; https://clinicaltrials.gov/ct2/show/NCT03134833 (Archived by WebCite at http://www.webcitation.org/76el0Viw9)

**International Registered Report Identifier (IRRID):**

DERR1-10.2196/11165

## Introduction

### Background

America’s HIV epidemic among youth aged 12 to 24 years has significantly increased in the last 15 years [1,2]. Young people now represent 26% of the HIV epidemic [1,2] despite investments in evidence-based behavioral interventions (EBI) and more recent scale-up of innovative antiretroviral treatments that can stop acquisition of HIV, which are known as preexposure prophylaxis (PrEP) and postexposure prophylaxis (PEP) [3,4]. Adolescents continue to become infected at disproportional rates [4]. It is critical to intervene with youth at high risk (YAHR) of acquiring HIV before they become infected. This study aims to intervene with YAHR with a set of interventions, which could be easily mounted, tailored, adapted over time, and broadly disseminated.

YAHR are those in urban epicenters and increasingly in the southeastern United States, particularly men who have sex with men (MSM) and transgender youth [1]. Homeless youth are also at elevated HIV risk, yet the last HIV seroprevalence study was conducted in 1991, showing 5.3% prevalence [5]. Youth in the criminal justice system may also be at elevated HIV risk [6,7]. YAHR are difficult to identify and intervene with in medical clinics because most youth (about 60% of general adolescent population) do not access health care [8-10]. Adolescents typically fail to disclose their sexual behaviors to their families or their physicians, most often because they are never asked about risk [11-13]. YAHR are likely to be encountered at agencies serving lesbian, gay, bisexual, transgender, and queer (LGBTQ) youth; homeless shelters; in the criminal justice system; and through in-person or Web spaces associated with sexual networking [14]. In each geographic epicenter of HIV, African American and Latino youth are at the highest relative risk of contracting HIV [1,15]. YAHR also typically seek peers, economic opportunities, and social services in the neighborhoods associated with the highest prevalence of HIV such as Hollywood in Los Angeles and the French Quarter in New Orleans [16]. This study uses community-based recruitment and screening to identify YAHR (see details in Rotheram-Borus et al [17]).

The current paradigm for reaching global HIV prevention goals (ie, increased uptake of the HIV prevention continuum [18]) is far more complex today than it was in the first 25 years of the epidemic. The US Centers for Disease Control and Prevention (CDC) recommends repeat and routine testing for HIV and sexually transmitted infection (STI) for YAHR every 3 or 6 months, as well as concurrently linking youth to HIV prevention and health care services and retaining them in care over time [19]. This study aims to operationalize and evaluate the impact of these guidelines using community-based recruitment and implementation of rapid diagnostic testing, referral and linkage to services, and easily scalable and tailorable technology-mediated interventions. The possibility now exists to implement biomedical and combination biobehavioral prevention for YAHR, which requires that youth know their serostatus (ie, be repeatedly tested for HIV over time), be linked to medical care, and consistently adhere to a strategy to protect themselves from HIV (eg, high adherence to PrEP, PEP, and condom use [18]). Textbox 1 summarizes the multiple endpoints for operationalizing uptake of HIV prevention continuum. These are particularly challenging tasks because the developmental challenges of youth evolve with age and may be more difficult for gay, bisexual, and transgender youth; homeless youth; and youth involved in criminal justice because of discrimination and stigma, which are often exacerbated for African American and Latino youth [11,20-23].

**The HIV prevention continuum for seronegative high-risk youth.**

- Test negative for HIV
- Receive health care twice annually
- Adherence consistently to prevention options
  - Preexposure prophylaxis or
  - Postexposure prophylaxis after condomless sexual encounters or
  - Condom use
- Repeat HIV and sexually transmitted infection testing 3 times annually

### A New Model for Intervention Design and Delivery

Traditional EBIs for HIV prevention rely on highly scripted and manualized protocols, delivered in-person, and that are time limited and became increasingly brief in numbers of and durations of sessions over the past 20 years [24]. Although EBIs for youth HIV prevention have been selected and diffused by the CDC on the basis of demonstrated efficacy in trials, they have been challenging to scale up and lack evidence for effectiveness [24]. These EBIs are highly structured and scripted manual-based protocols, which have been noted as being difficult to implement with fidelity and are not tailorable to intervention facilitators’ and youths’ varying styles, preferences, hierarchies of needs, development stages, and HIV risks. They also typically rely on in-person visits at community-based organizations (CBOs) or health care settings. Disruptive innovations of massively scalable mobile and social media technologies may be able to implement and broadly reach youth with prevention messages and linkage to services [24-26]. Bower and Christensen [27] defined the concept of disruptive innovations in *Harvard Business Review* as simpler, cheaper, and *good enough* solutions to meet the majority of consumers’ needs and preferences relative to incumbent products and services that are often designed for the highest-need consumers, for example, ATM machines versus bank tellers, US $2 reading glasses versus specialized prescriptions, or minute clinics in pharmacies. Rotheram et al (2012) applied this concept to EBIs for youth behavioral and mental health problems by positing that interventions based on common practice elements identified across manualized EBIs, and technology-mediated modalities could be disruptive innovations that might be more scalable, adaptable, and amenable to providers’ and patients’ needs and preferences [24].

Advances in mobile phone and social media technologies have created opportunities to engage and intervene with large numbers of youth at relatively low costs, using technologies that permeate their daily routines [25,28]. This study will use 2 primary technology platforms, that is, text messaging and social media, in addition to telephone and in-person visits based on youths’ preferences. Text messaging, email, and social media use are nearly universal among youth [29-31]. Approximately 90% of youth report having a mobile phone [31-33], 90% of them text about 30 times each day [31]; rates are similar for homeless youth but with some inconsistency in maintaining service [29,30,34]. Similarly, over 90% of youth access the Web daily, and smartphones have become the primary mode of accessing the internet for the majority of youth [31].

This study’s automated and interpersonally delivered technology-mediated interventions are based on the shared features of existing EBI—we do not aim to create a new smartphone app or an EBI with a manual to be *replicated with fidelity* [35,36]. In the last 25 years, over 100 HIV EBIs and 36 adolescent sexual health EBIs have been identified by the CDC and other review bodies as efficacious [37-39] and supported for diffusion. Members of this study team rated the manuals of 5 of the CDC’s most popular, behaviorally oriented primary prevention EBIs for adolescents [36,40,41,42], finding that each of them incorporated common processes, principles, and factors. Each of the EBIs are also based on cognitive behavioral theories, even though some researchers cited a more specific iteration (eg, social cognitive theory, theory of reasoned action, and the AIDS risk reduction model) or meta-theory (eg, information-motivation-behavior and social action theory). The EBIs had much more in common than different [36,40-42]. The intervention approach for this study is to focus on the common elements that many different interventions share, delivered by the most cost-efficient, scalable, and adaptable delivery strategies that permeate youths’ daily routines: mobile and social media technologies.

This hybrid implementation-effectiveness study examines alternative models for implementing the CDC’s guidelines for routine HIV/STI testing for YAHR of acquiring HIV and for delivering EBIs in modular elements instead of scripted manuals, while also monitoring implementation, costs, and effectiveness [43]. A randomized controlled trial (RCT) evaluates the efficacy and cost-effectiveness of the 4 intervention strategies of variable intensities and costs. This study aims to inform future prevention programs implemented by communities to avert the acquisition of HIV among young people by monitoring outcomes at 4-month intervals over 24 months.

## Methods

### Overview

All procedures in this study have been approved by the institutional review board (IRB) of the University of California, Los Angeles, which serves as the single IRB of Record for researchers at collaborating institutions. This is 1 of 3 studies in a National Institute of Child Health and Human Development (NICHD)-funded U19 Cooperative Agreement for the Adolescent (HIV Medicine) Trials Network (ATN) as well as a Management Core and Analytic Core [17,44-47]. This study began recruitment in May 2017. From June 2018 to December 2018, the protocol underwent a process of review and revision in collaboration with NICHD project scientists, the study’s scientific monitoring committee, and an external statistical expert, to meet budget constraints and align more closely with ATN scientific priorities. The major changes were (1) to reduce STI testing frequency from every 4 months routinely to only at baseline, 12, and 24 months and rectal testing only (unless requested by youth or indicated by symptoms); (2) stopping follow-up and intervention at 12 months with youth who are not MSM or transgender; and (3) changing randomization allocations for person-mediated intervention arms to have statistical power for MSM and transgender participants with 70% retention rates.

### Design

**Figure 1:**
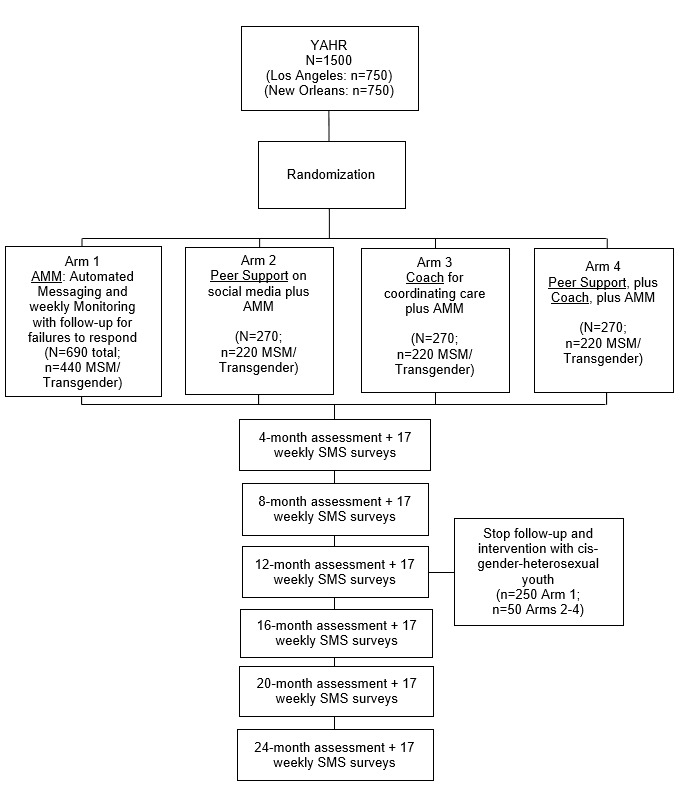
Design of the randomized controlled trial for youth at high risk (YAHR) for acquiring HIV (n=1500). AMM: automated text-messaging and monitoring; MSM: men who have sex with men; SMS: short messaging service.

Figure 1 shows the study design overview. The randomized controlled factorial design assesses efficacy of 3 intervention strategies (arms 1-3) and their combination (arm 4). The person-mediated interventions in arms 2 to 4 have a sample size of 270 per arm with 220 MSM and transgender youth, whereas the larger sample of 690 in the automated intervention only (arm 1) is because of the broader U19 Cooperative Agreement’s goal to identify youth acquiring HIV infection during follow-up to refer to a sister protocol on acute HIV infection [45], and earlier expectations to recruit youth that could be referred to other ATN protocols. Participants are followed longitudinally over 24 months (12 months for youth who are not MSM or transgender) and assessed at 4-month intervals by interviewers. Participants also complete a brief 7-question survey every week by text message (or email when nonresponsive to text messages). The overviews of assessments are provided below and described in detail in linked publications for the U19 [17,44-47].

### Recruitment

In both Los Angeles and New Orleans, the Recruitment, Engagement, and Retention Centers in the Management Core [17] are responsible for recruiting, enrolling, and following up with youth. Youth are primarily recruited from CBOs and clinics serving gay, bisexual, and transgender youth; homeless youth; and youth on probation or released from incarceration. Youth are also recruited through dating apps (eg, Grindr, Jack’d, and Scruff), including while present in social venues (eg, bars, clubs, and community events). We anticipate the study sample to be predominantly MSM and transgender, African American and Latino, and aged between 18 and 24 years. Youth are approached with verbal assent to complete brief screener questions and a rapid HIV test; however, youth aged between 12 and 14 years are asked to provide written voluntary informed consent to screen because of IRB requirements. Details on recruitment and screening are provided in other publications from the U19 [17,47] and briefly summarized below.

### Eligibility

To screen as eligible for enrollment, youth must test seronegative on a rapid HIV testing at screening and report at least 3 of the following criteria: self-reporting as gay, bisexual, or transgender; African American or Latino race/ethnicity; having unprotected anal sex, sharing needles for injecting drugs, or an HIV-positive partner in the last 12 months; having been homeless (defined as not having a regular place to sleep for 3 or more months); illicit substance use (not including marijuana) in the last 12 months; having been hospitalized for a mental health disorder; having been in jail or on probation; having an STI in the last 12 months. Transgender and MSM youth are always eligible. Eligible participants are invited to enroll in the study with written informed consent.

### Assessments

Following enrollment, participants complete a baseline assessment, which includes a questionnaire and a series of rapid diagnostic tests. These assessments are repeated at follow-ups. Participants receive a US $50 cash incentive for each baseline and 4-month follow-up assessment completed. Other assessments include weekly monitoring surveys, staff implementation time and processes, and costing data for cost-effectiveness analyses. These assessments are briefly described below and in detail in Rotheram-Borus et al [17].

#### Rapid Diagnostic Tests

The following rapid diagnostic tests are performed (see Shannon et al for details [47]):

1. HIV—Rapid test: Clinical Laboratory Improvement Amendments (CLIA)-waived Alere Determine HIV-1/2 Ag/Ab Combo fingerstick blood test for HIV-1/2 antibodies and the HIV-1 p24 antigen with a detection window of 12 to 26 days; results are available in 20 to 30 min. Once enrolled, potential acute HIV infection is assessed in batches using the Cepheid Xpert HIV-1 Qual Assay to detect HIV-1 total nucleic acids for acute HIV infection, or lab-based polymerase chain reaction (PCR) testing.
2. Chlamydia and gonorrhea—Food and Drug Administration–approved Cepheid Xpert CT/NG Assay PCR test using vaginal swabs from women, urine samples from men, and pharyngeal and rectal swabs from both women and men. Results are available in 90 min.
3. Syphilis—CLIA-waived Syphilis Health Check fingerstick blood test to detect treponemal antibodies with a 10- to 15-min time to completion.
4. Substance use—A multidrug urine test panel to detect the presence of marijuana, cocaine, opiates, and methamphetamine with result available in 2 to 5 min.
5. Alcohol use—The BACtrack breathalyzer to determine blood alcohol content over the past 48 hours.

Any study participants who test positive for HIV are immediately linked to care for treatment and offered enrollment in one of the ATN Comprehensive Adolescent Research and Engagement Studies (CARES) protocols for youth living with HIV, depending on stage of HIV infection determined by Fiebig stages 1 to 6 on HIV-1 antibody using Western blot test [45]. Participants testing positive for other STIs are provided immediate treatment by the study team, including partner therapy.

### Questionnaires

Questionnaires are administered by an interviewer in a private room at partner sites or study offices, using Android tablets and take approximately 45 min to complete (see Rotheram-Borus et al for details [17]). Briefly, baseline questions cover lifetime and past 4 months; follow-up assessments cover the past 4 months. Questionnaires assess sociodemographic factors; health care access, insurance, and utilization; substance use; sexual behaviors; PrEP and PEP use; mental health; social media use; and locator information and consent to access medical records and to use social security numbers for locating efforts. Interviewers enter the participants’ responses and rapid diagnostic test results in the CommCare mobile-Web electronic assessment and case management system that is cloud based and Health Insurance Portability and Accountability Act (HIPAA) compliant, by Dimagi Inc (see Comulada et al for details [44]). CommCare also manages and sends intervention and assessment text messages as part of the automated text-messaging and monitoring intervention (AMM).

### Weekly Monitoring Surveys

The monitoring strategy is based on a weekly *Check-In* survey, which can be completed via text message or a Web link sent via email with a HIPAA–compliant RedCap version of the survey. Surveys assess the previous week time period with 7 questions on potential symptoms of acute HIV infection (yes/no), STI symptoms (yes/no), and number of days of feeling sad/depressed, sex without condoms, drug or alcohol use, not having a place to sleep or enough food; and miss taking medications (if taking medications). Participants receive a US $1 incentive per weekly survey completed, either in cash at their next 4-month follow-up assessment or on demand, including via electronic transfer (via Paypal, Venmo, or Zelle). Yes responses to HIV and STI symptom questions are reported to interviewers for follow-up risk assessment and rapid testing within 2 weeks. Reports on sadness/depression are monitored for potential follow-up and referral.

### Outcome Measures

Our primary outcome is uptake and adherence to the HIV prevention continuum, according to the following measures, which will be analyzed individually and in sum (see Statistical Methods below):

1. Linkage to medical care reflected in a visit twice annually, at a minimum, to a health care provider
2. Consistent utilization of condoms, PrEP, or PEP
3. Participation in other HIV prevention programs and services
4. Repeat assessments for HIV and STI testing 3 times annually

Secondary outcomes include mental health symptoms, substance use, and housing security, which are hypothesized to impact primary outcomes as mediators or moderators of intervention effects.

### Costing and Implementation Data

There are 2 types of costs: costs of delivering the intervention and the additional costs incurred by participants for their use of health care services and services from other agencies (eg, use of health care services). Intervention costs are classified into 1 of 4 categories [48]: (1) capital equipment (eg, computers); (2) recurring supplies and services; (3) facility space; and (4) personnel, including fringe benefits. Costs in the first 3 categories are obtained from project records. Personnel costs include hours and wages of staff to design and deliver the interventions, including peers, coaches, supervisors, facility charges, software costs, and short messaging service (SMS) and other social media costs, messaging and mobile app data costs, additional time in coaching and supervision, and server hosting. Personnel time is estimated from time reported on time sheets for hourly employees and budgeted time for other staff and investigators. Time spent on specific activities for hourly staff (eg, coaches and interviewers) is assessed in detail over 1-week periods quarterly using the Time It app [49] on their study-issued Android smartphones. Recorded time over 1-week periods is extrapolated to cover total time over the study period. The costs of additional services are derived from respondent reports on utilization and medical records and are estimated using publicly available data. Research-specific costs, such as incentive payments, informed consent, assessments, and software adaptation for survey tools, are excluded from total costs. All cost data are price-adjusted back to the first year of the study, using the medical care component of the consumer price index.

### Intervention Development

#### Youth Advisory Boards

Consistent with the model of community-based participatory research [50,51] and requirements for all ATN studies, YABs reviewed and provided feedback on all study protocols and interventions before study launch and are involved on an ongoing basis to ensure that interventions are continuously improved. In particular, YABs reviewed and provided feedback on adaptations or cutting of every text message in the libraries of existing text messages (details below). YABs also provide topics of interest for peer support discussion boards and its associated website content and branding. YABs comprise about 10 seronegative YAHR and youth living with HIV (undisclosed) in both Los Angeles and New Orleans and reflect the diversity of the youth in both cities.

#### Text-Message Libraries

The existing libraries of HIV prevention messages adapted by the YAB include: (1) Project Tech Support [52], which has developed over 600 theory-based text messages specifically for methamphetamine-using MSM focused on reducing sexual risk behaviors and methamphetamine use and increasing ART use and adherence for those who are living with HIV; (2) the UCARE4LIFE text-message library from the Health Resources & Services Administration HIV/AIDS Bureau [53], which were designed for youth living with HIV but adapted for YAHR for this study; and (3) the PrEPTech library from youth+tech+health focused on increasing uptake and adherence to PrEP [54]. These libraries formed the basis of the initial text-message content for adaptation in collaboration with the YABs. Message libraries have been tailored for 2 different risk profiles—LGBTQ and heterosexually identified youth. Research indicates that messaging interventions based on cognitive behavioral theory are more likely to be successful [52,55-57]. In particular, text-message libraries from Project Tech Support [52], which form the majority of messages adopted for this project, were based on Social Support Theory [58-60], the Health Belief Model [61-63], and Social Cognitive Theory [64,65].

### Intervention Conditions to Optimize the HIV Prevention Continuum

#### Condition A/Arm 1: Automated Text-Messaging and Monitoring Alone

AMM is a relatively low-cost and scalable intervention that could be diffused nationally. AMM is provided to all study participants across study arms as part of the enhanced standard of care and ethical requirement to provide prevention information to high-risk youth per ATN guidelines.

##### Daily Texts to Inform, Motivate, and Refer Youth to Services

Messages are sent daily, at times selected by each participant. Some evidence suggests that several text messages each day might be required to have an impact on behavioral outcomes [66-68]; therefore, up to 5 messages are sent per day in 5 content streams outlined in Table 1. Participants may opt-out of and opt-in to each of the 5 message streams at any time during the study by contacting interviewers and by updating preferences at each follow-up assessment when interviewers prompt participants and collect feedback on message experience. If a participant texts *STOP*, the SMS gateway provider (Twilio) stops sending all text messages. Messages are sent every day on health care (eg, medical, dental, and provider interactions), wellness (eg, mental health, diet and physical activity, social support, housing, jobs, and education), and medication reminders (if taking). Messages on sexual health and substance use are sent on Thursdays, Fridays, and Saturdays, a design decision based on YAB guidance to minimize messaging burden for these sensitive topics while maximizing impact on days when risk behaviors are most likely.

**Table 1:** Automated text-messaging and monitoring intervention daily text message examples.

Text message type and Risk Profile: MSM^a^		Risk Profile: non-MSM
**Health care (70 messages)**		
	Your health is important.	First things first. Are you doing everything you can to stay healthy?
	Vaccinations can be injections, drops or sprays. They are a proven way to prevent disease and keep you healthy.	It's your life we're talking about. Be a part of EVERY decision about your health care.
	New in the area? Make sure to get a new doctor close by! Go to http://tinyurl.com/js8mqa6 to find free clinics close to you.	Get nervous talking to your provider? Write down any questions you have and bring them with you so you don’t forget.
**Wellness (70 messages)**		
	Friends can be good medicine. If you need to talk, give a friend a call.	Have you laughed today? Laughing is a great form of stress-relief, get some laughs in your day!
	Gay Pride is taking care of yourself.	It's OK to ask for help.
	Been inside all day? Get outside and soak up some quick sun for a boost of energy.	A budget can help make sure you have enough money every month. To learn more, visit http://tinyurl.com/kx8bxp2
**Sexual health (100 messages)**		
	If your partner wants to get tested for HIV, text KNOWIT (566948) and enter their ZIP code. KNOWIT will text back a nearby testing site.	Left untreated, some STIs can cause health problems that make it hard or impossible for a woman to get pregnant. Visit http://1.usa.gov/1dm9P0B to learn more.
	Open relationship? Know your boundaries.	Make sure the only thing you “get” is laid.
	Friction is the enemy. You can lube up every time.	Myth: Women can’t give men HIV; Fact: Both men and women can get HIV from vaginal and anal sex.
**Substance use (90 messages)**		
	Stay in control—people who are drunk or high take more risks.	When was the last time you had sex sober?
	Drinking alcohol can take a toll on your body. Take care of you!	Only take a fixed amount of cash out (and no cards) if you want to control how much you drink.
	Spending too much money on Tina?	Going out tonight? Be safe. Party smart.
**Medication adherence (100 messages)**		
	Reminder. It's going to be a great day.	It's that time again.
	When you take your meds regularly, you're in control.	Take care of yourself today.
	Is your stomach feeling a little off after taking your PrEP? Try taking your pill with food to ease possible stomach discomfort.	Where are you storing your PrEP? Your hot car or fridge can damage the medication-- keep it at room temperature.

^a^MSM: men who have sex with men.

##### Weekly Monitoring

In addition to monitoring for HIV/STI symptoms for follow-up testing and linkage as described above for study assessments, monitoring also functions as self-monitoring. Self-monitoring is a key skill for self-management and a core construct in social cognitive theories [69-71]. Preliminary studies on mobile self-monitoring demonstrated feasibility and acceptability [72,73], validity and reliability [74,75], compliance (ie, protocol adherence) [76], and user preferences. The efficacy of theory-based mobile self-monitoring to support self-directed self-management has also been demonstrated [74,77-80]. Self-monitoring by mobile or Web apps to support motivational interviewing to reduce substance use and sexual risk has demonstrated efficacy with substance users, persons living with HIV, and persons at high-risk for HIV infection [81-86]. In this study, weekly text-messaging monitoring surveys remain open for response for 48 hours. In the cases of nonresponse, CommCare automatically sends a follow-up prompt 24 hours after the initial prompt. After 2 weeks of nonresponse, interviewers initiate follow-up to assess current status of the youth. If participants are nonresponsive to text messages or opted-out via the SMS gateway by texting STOP, emails are sent instead with a weblink to a RedCap version of the survey.

#### Condition B/Arm 2: Automated Text-Messaging and Monitoring and Online Peer Support Groups Via Private Social Media

Peer Support groups are a low-cost strategy to enhance prevention and adherence interventions. Relationships have been shown to be motivating and increase engagement and retention in care for a range of chronic diseases [87-89]. Adolescence, in particular, is a developmental period where the influence of peers is crucial [90]. Almost every EBI for HIV prevention in the CDC’s Compendium of EBI has a peer support component [91]. Several studies or online peer support groups with young minority MSM and LGBTQ youth have demonstrated preliminary efficacy for reducing HIV risks [92-94]. In addition, 2 other online peer support group interventions combined with peer paraprofessional coaches via private Facebook groups (no longer feasible because of privacy and IRB concerns) found increased requests by MSM for HIV home test kits in Los Angeles [95] and increased clinic-based HIV testing in Peru [96]. Although a recent meta-analysis of 31 RCTs did not find significant benefits for electronic peer-to-peer interventions alone [97], the review noted that studies combining peer support with other interventions found some evidence for efficacy on the basis of associations between greater use of peer support via social media, indicating a dose-response association [98-100]. Therefore, this study is examining online peer support groups, moderated by paraprofessional near-peer coaches, in conjunction with AMM and also in conjunction with coaching in study Condition D/Arm 4.

Participants randomized to online peer support groups (Conditions B/Arm 2 and D/Arm 4) are invited to participate in a private Web discussion board hosted on Muut. Muut is an open-source discussion platform that is mobile- and desktop-friendly. Users can personalize their Muut profiles using avatars and photos, and content created can be continually reorganized according to new and relevant *channels* (eg, for a PrEP channel and mental health-related channels). Muut includes social media features such as *likes* and emojis, and multimedia content by embedding the forums in the study website. Private messaging functions are disabled because of IRB concerns around communication among participants that cannot be monitored or moderated. Participants are required to register and request access to join, which is facilitated by detailed screenshot instructions sent by SMS and in-person by coaches at the recruitment sites. Coaches and project coordinators review access requests to ensure that only study participants are attempting to join the forums and that their usernames do not compromise their anonymity by including their names. In total, 2 forums are available, 1 for LGBTQ-identified youth and another for heterosexual-identified youth.

Coaches and intervention coordinators seed discussions by creating and posting blogs, polls, and discussion topics twice a week on popular culture and general health and wellness to increase engagement (on the basis of YAB feedback), in addition to HIV prevention continuum themes (health care, PrEP, PEP, condom use, STIs, and HIV) and secondary outcome themes (mental health, substance use, and housing), including referral, resource, and services information. Coaches and coordinators also moderate the forums throughout each day to ensure that ground rules are followed, delete inappropriate posts, post correct information, and engage with and reward participant-initiated content. Participants are given warnings and removed from the discussion board if they post inappropriate content 3 times after receiving feedback for each occurrence, which includes: solicitations for sex and drug use; racist, homophobic, or other stigmatizing content; pornographic content; *trolling* inflammatory remarks or personal insults.

Participants are incentivized to participate and support their peers by posting questions and new discussion threads and responding to content posted by peers and coaches, such as sharing experiences and advice. Participants receive US $10 in cash or electronic transfer for initiating or responding to posts 3 times in a week, for up to 16 weeks over follow-up period.

#### Condition C/Arm 3: Automated Text-Messaging and Monitoring and Coaching—Strengths-Based, Youth-Centered

There are 2 levels of coaching engagement and overall functions in this intervention condition anticipating youths’ varying preferences and needs over time; patient navigation (ie, services and resource referral and linkage) and more intensive, strengths-based, youth-centered, goal-focused coaching. *Patient Navigators* are one of the primary strategies advocated to link and retain high-risk populations to prevention and treatment services. Similar to navigators used for chronic diseases [101-103], the CDC recommends that patient navigators can help optimize the HIV prevention continuum [104,105]. Patient navigation involves a paraprofessional or experienced peer helping persons link to health care and services, assist with insurance, problem solve barriers to care, and provide supportive counseling and follow-up to motivate engagement and retention in health and prevention services. Coaching is based on the strengths-based model [106], which has demonstrated positive impact with homeless youth [107] and persons living with HIV [108]. Critical components of the model include identifying personal and interpersonal strengths rather than deficits and then setting, problem-solving, and accomplishing long- and short-term client-centered goals selected by participants in collaboration with coaches with a focus on hierarchies of needs (housing, food, and employment) as well as programmatic priorities.

In this study, coaching formally begins with a strengths-based assessment, an approximately 45-min open-ended interview that addresses 6 life domains: (1) daily living (survival needs such as food, housing, finances, and employment); (2) physical health (non-HIV related health problems); (3) health care; (4) social relationships (including social support, disclosure, and stigma); (5) mental health; and (6) HIV risks (substance use and risky sexual behaviors). Youth are asked to identify their current status within each domain, as well as strengths and challenges in each area. This assessment guides the development of personalized goals. Each youth has a maximum of 3 goals at any given time. The coach and youth identify a primary goal to address following the session including identification of resources and skills needed to achieve the goal (eg, problem-solving and coping skills). Typically, long-term or lofty goals must be broken down into smaller short-term *SMART* goals (ie, specific, measurable, achievable, realistic, and timely). Responsibility for goals is shared among the youth and coach depending on the nature of the goals. At each subsequent session, the coach *checks in* with the youth on goals set in previous sessions. As goals are accomplished, new goals are set. Goals not met are problem solved and adjusted to be achievable in successive approximation.

Coaches focus on the following priorities in their contact with youth:

1. Crisis support to address youths’ immediate priorities and needs, particularly housing, which are typical barriers to engaging in other health-promoting activities
2. Completion of a strengths-based assessment session, including goal setting
3. Problem-solving priorities and facilitating linkages to prevention services, health care, and other services and providers (eg, Case Managers at recruitment sites, nearby agencies, or providers for mental health, substance abuse, housing, jobs, school, in conjunction with Case Managers, if available)
4. Appointment coordination, scheduling, and reminders
5. Follow up with clients to give a rewarding message for attending appointments (eg, Great job attending your appointment!) or to problem solve barriers if patient missed an appointment

Although the ultimate aim of this study’s coaching intervention is to improve HIV prevention continuum, coaches also aim to address the hierarchy of needs and secondary outcomes that are hypothesized to influence prevention outcomes, such as homelessness, employment, mental health, and substance abuse. More details on the coaching intervention are provided in a publication for a sister protocol for youth living with HIV [47].

Coaching represents the most intensive person-mediated strategy in this study; however, coaches use a variety of means of communication and interaction on the basis of participant preferences and responsiveness. These include text messaging, phone calls, social media private messaging, video chat, email, and in-person contacts. In particular, in-person contacts are accommodated to meet youths’ preferences for initiating a coaching relationship and building trust and rapport, including on an ad hoc basis at recruitment sites with coaches being on site to engage participants who are nonresponsive to initial text message and telephone contacts.

Importantly, and as noted in the introduction, this is not a manualized or scripted intervention. Instead, it is based on training, monitoring, and supervision using common practice elements or skills identified across EBIs for youth prevention and behavioral health [109,110], in addition to the priority topic domains of the project. The practice elements used are engagement/rapport building (including setting expectations), goal setting, problem-solving, praise, self-monitoring, assertiveness communication, triggers, relaxation, social support networking, positive activities/alternatives, setting up rewards, positive self-talk, monitoring (by coaches), emotional regulation, relapse prevention, modeling/role-play, and referrals. The content domains are daily living (housing, food, and employment), social relationships, sexual behaviors, PrEP/PEP use, anxiety, depression, other mental health, substance abuse, physical health, violence, and crisis support. Training modules and monitoring tools are based on these practice elements and content domains.

After every contact with a participant, whether a full coaching session or brief navigation interaction or follow-up, the coach completes a brief interaction log or monitoring log using the CommCare mobile-Web app on smartphones, tablets, or Web-connected computers. The log forms record the practice elements used and content domains covered during the interaction. This activity logging functions to prompt coaches to use the skills and address the content priorities of the intervention while simultaneously providing fidelity monitoring to inform supervision and for data analyses.

##### Training and Supervision

Coaches are near-peer, bachelor’s-level paraprofessionals and of similar age, ethnicity, gender, and sexual identity to the participant populations. In addition to the components of the strengths-based model, practice elements, and content areas outlined above, coaches are taught the foundational theory of behavior change (people change slowly over time with small steps and with opportunities and rewards), the shared principles of behavior change (Be Prepared; Act on facts, not feelings). Coaches participate in weekly, cross-site supervision via in-person and videoconference meetings to debrief and jointly problem-solve logistical and clinical challenges with the principal investigator and supervisors. The practice elements are reviewed and reiterated during weekly training and learning community calls with all coaches as their toolbox for addressing the core content areas to be addressed. In both Los Angeles and New Orleans, there are local, on-call clinical psychologists for participants in crisis and who also provide weekly clinical supervision and ongoing booster training to coaches.

#### Condition D/Arm 4: Automated Text-Messaging and Monitoring and Peer Support Via Social Media and Coaching Automated Text-Messaging and Monitoring

This intervention condition delivers the combination of the above interventions, which enables estimation of the cumulative or synergistic effects of what might be considered an ideal model of support for high-risk seronegative youth to optimize their engagement and retention in the HIV prevention continuum.

##### Data Analysis

Analyses are described according to each of the study aims.

###### Aim 1: To Assess the Independent and Synergistic Effects of the Interventions on the HIV Prevention Continuum Outcomes

Multilevel models (MLMs) will be used to test the impact of the intervention on HIV prevention continuum indicators and secondary outcomes over time shown in Textbox 1 in the Background section. MLMs are needed to account for the hierarchical nature of the data and model correlations between repeated observations to properly estimate standard errors on regression coefficients. MLMs are flexible in handling discrete outcomes, such as binary HIV-prevention-continuum indicators (yes/no) and continuous outcomes, such as mental health measures. The MLM analyses will contain main effects for peer support (PEER_i_) and coaching (COACH_i_), as well as a 2-way interaction between peer support and coaching compared with AMM alone. This model parameterization will allow us to test independent effects of peer support and coaching and their synergistic effects on outcomes. MLMs contain interactions between TIME and intervention effects to test for changes in outcome levels between intervention arms over time (our primary goal). Equation 1 shows a random intercept (RI) model that will provide a starting point in the modeling process. Let Y_it_ be an outcome for person i at time point t and let η_it_ be a link function for outcome Y_it_, such as a logit link for binary prevention-continuum indicators. An MLM with a random effect λ_i_ to capture correlations between repeated observations for each person is expressed as:

η_it_ = β_0_ + β_1_ PEER_i_ + β_2_ COACH_i_ + β_3_ TIME_it_ + β_4_(PEER_i_ x TIME_it_) + β_5_(COACH_i_ x TIME_it_) + β_6_(PEER_i_ x COACH_i_ x TIME_it_) + λ_i_. (1)

We will also fit MLM with other covariance structures that we have used in previous HIV intervention studies, including RI and slope (RIAS) and autoregressive covariance structures. The covariance structure with the best fit statistics will be selected. Covariates for demographics and other background characteristics may need to be added to Equation (1) if imbalances are found across intervention arms at baseline.

As a first step, MLM will be fit to each primary outcome and secondary outcome and intervention effects for each outcome will be evaluated separately. We will also evaluate the overall impact of the intervention across binary indicators for optimization of the prevention continuum utilizing a strategy employed by this team to analyze multiple outcomes with one overall statistic, to reflect if there is an overall impact on multiple binary outcomes [111]. Analysis of multiple outcomes through separate regressions increases the probability of finding a significant intervention effect by chance (ie, type I error is inflated). Therefore, we will properly adjust the type I error by conducting simulation studies to determine how many significant intervention effects are needed to declare an effective intervention. Simulation studies assume binary outcomes to be correlated to model a real-world phenomenon.

###### Aim 2: To Assess the Temporal Relationships Between the Primary and Secondary Outcomes

The temporal relationships between primary and secondary outcomes are analyzed using a bivariate-outcome MLM to examine bidirectional relationships between primary outcome and secondary outcome observations at different time points. One parameterization of the bivariate-outcome MLM that we have used in a previous HIV study to examine the time-varying relationship between HIV-transmission behaviors and mental health symptoms is the bivariate RIAS model [112]. This model is formulated through 2 separate MLM equations for each outcome, k=1,2, that are linked through random effects to model RIs λ_0ki_ and slopes λ_1ki_. A covariance matrix is also modeled that includes correlations between random effects λ_0ki_ and λ_1ki_. Correlation between random effects captures time-varying associations between outcomes, such as the correlation between the first outcome at baseline and the second outcome over time, and vice versa. Building off on Equation (1), the basic bivariate RIAS model is expressed as:

η_kit_ = β_0_ + β_1_TIME_kit_ + λ_0ki_ + λ_1ki_TIME_kit_. (2)

###### Aim 3: To Assess the Relative Cost-Effectiveness of the Interventions

The deployment of all HIV prevention strategies today must be based on the cost-effectiveness of peer support and coaching to automated messaging for HIV prevention continuum outcomes and reducing risk behaviors, substance use, and mental health problems. The cost-effectiveness analysis will compare the additional cost required, on average, to get an additional unit of outcome in the 2 person-mediated interventions (peer support and coaching) and in the attentional control (automated messaging) by calculating a cost-effectiveness ratio (CER) [113]. The CER is the difference in total costs of providing a person-mediated intervention versus automated divided by the difference in person-mediated outcome and automated outcome [113]. Primary outcomes of HIV prevention continuum and secondarily substance use and mental health are outcomes of interest. Costs are measured as:

CER = (C_person_ − C_auto_) / (O_person_ − O_auto_). (3)

Analogous CERs will be calculated for peer support versus automated/attentional control, patient coaches versus automated, and for the combined peer support and coach versus automated control. CERs will be calculated at final the follow-up. We expect the person-mediated interventions to incur greater personnel costs than the automated ones. On the other hand, the person-mediated interventions may result in greater use of other mental health or drug treatment services than the automated group. These greater costs may or may not be offset by reduced costs of other services, such as incarceration, relative to the person-mediated groups. The CER answers the question of whether improvements in outcomes are worth any added costs. If the person-mediated interventions result in both better outcomes and lower net costs, it will be deemed *cost-saving*.

We conduct sensitivity analyses, as recommended by Gold [113] to estimate the extent to which the CER calculation is affected by differences in assumptions about the size of the differences in treatment effect. In particular, we determine how sensitive the CER is to assumptions that the difference in treatment effect is 1 SD below or above the mean estimated effect size. Similarly, we estimate the sensitivity of conclusions to costs that are 1 SD below or above the estimated mean.

##### Sample Size Calculations

Sample size calculations are conducted to detect changes in the probability of an HIV care continuum yes-no indicator, such as PrEP adherence, STI treatment, and 100% condom usage, over 7 time points (every 4 months over 2 years) for the MSM/transgender sample. Calculations show that we have at least 80% power to detect differences in the probability of an indicator as small as 10% to 16% at the last time point between 2 arms with 220 participants at 70% retention. Sample size calculations were conducted through simulation using the following steps. *First*, binary indicator values were simulated from a binomial distribution with the probability of an event (yes) on the basis of an MLM similar to Equation 1. Simulation regression coefficients were specified with baseline rates of 20%, 50%, and 80% to cover a range of care continuum rates we have encountered in previous HIV research and were set to be the same between intervention arms. We specified normally distributed random effects as we did in Equation 1 with an SD of 1.5, similar to what we have found in other studies. Finally, we assumed 30% loss to follow-up and used a sample size of 160 in each arm in simulations. In practice, we anticipated a much lower attrition rate but wanted to be conservative in our sample size calculations. We simulated 1000 datasets for each of the baseline testing rates we specified and for different sample sizes for 2-arm comparisons. *Second*, we fit MLM models to each of the 1000 simulated datasets for differing combinations of parameters. *Finally*, power was estimated to be the ratio of the number of MLM with a significant difference between intervention arms over time divided by 1000. In the end, exploratory analyses will be conducted using the same analysis plan outlined above for 12-month follow-up that includes the cisgender-heterosexual participants.

## Results

The project was funded in September 2016, and enrollment began in May 2017. Enrollment will be completed between June and August 2019. Data analysis is currently underway, and the first results are expected to be submitted for publication in 2019.

## Discussion

### Summary

The goal of this study is to test the efficacy and examine the cost-effectiveness and implementation of alternative models for delivering the CDC’s guidelines for routine HIV/STI testing for YAHR of acquiring HIV and for delivering evidence-based interventions in modular elements instead of scripted manuals and in flexible technology-based delivery formats. The technology-mediated interventions for YAHR in this study aim to improve HIV prevention continuum of engaging in medical care, adopting PEP after HIV exposure or PrEP before HIV exposure, or a behavioral protection strategy, as well as repeatedly testing for HIV and STIs. Consistent access and utilization of medical care is a common challenge for adolescents and young adults [114], particularly African American and Latino youth [115]. Text messaging and social media technologies offer relatively low-cost modalities to scale interventions for adolescents nationally. The study design provides opportunities to assess the efficacy, potential synergistic or cumulative effects, and cost-effectiveness of the proposed automated and person-mediated strategies. The study assessments will also enable examination of time trends in onset and periodicity of risk, and the relationships between primary and secondary outcomes in bivariate outcome analyses. In the evaluation of each intervention condition’s cost-effectiveness for the primary outcomes, we hypothesize that each intervention of increasing intensity (AMM, online peer support groups, and coaching) will have greater efficacy but that the added costs may not justify use at scale.

The mobile and social media intervention arms in this study build off the relatively nascent evidence base of internet, mobile, and social media interventions for populations at high-risk for acquiring or transmitting HIV [116-120]. Although technology-based assessments (eg, Web- and text-message surveys) [92,117] have demonstrated success with large samples in the thousands [121], mobile and social media technology intervention studies have tended to be in small scale by comparison [122]. Although several large-scale RCTs are currently underway to address HIV, most focus on a single technology-based strategy or a bundle of strategies in a single intervention arm, instead of a comparison of multiple strategies as in this study [120,123,124].

Youth present with a wide variety of issues that affect their risk for HIV infection. In addition, they can be extremely labile in terms of their emotional and behavioral reactions to life events as they undergo developmental changes, which affect their risk. The interventions in this study are available to youth over the duration of their study follow-up of 24-months for YAHR of acquiring HIV in the US (ie, MSM and Transgender youth) and 12-month of follow-up for other youth at elevated risk. This approach acknowledges variability and unpredictability of risk behaviors as adolescents experience developmental transitions. The interventions are available to prepare youth for transitions or to be available when transitions, crises, and risks occur. This is in novel contrast the last 30 years of the EBI movement for HIV prevention, which incentivized highly structured and increasingly brief interventions that demonstrated only short-term impacts on behaviors.

### Strengths and Limitations

A limitation of this study is that study procedures and enhanced standard of care are relatively intensive interventions in and of themselves. Routine or indicated HIV and STI testing several times per year follows the CDC’s recommendations for people at high risk of acquiring HIV; however, this study operationalizes this with active and incentivized follow-up, community-based testing, and immediate treatment provision and partner therapy for STIs without a clinical visit. Daily text messaging and weekly monitoring with follow-up add another layer of intervention that cannot be directly assessed in this study. These limitations result from several factors, including utility for study assessments, ethical standards for research with vulnerable adolescents and the ATN to provide some base level of prevention support, goals for the broader U19 to identify youth with acute HIV infection from this study’s participants over time for a sister protocol [45], and to operationalize and examine costs and impacts of implementing CDC guidelines with community-based strategies [125]. Therefore, the potential efficacy and cost-effectiveness of the online peer support group and coaching interventions examined in this study should be considered within this context.

### Conclusions

The study findings will be invaluable to inform future adolescent prevention interventions, not only for HIV but also in many other areas. This study will have policy implications for the allocation of resources to HIV testing resources in local communities, the uptake and scalability of interventions for youth, and innovative approaches for designing and diffusing EBI globally.

## Abbreviations

AMM: automated text-messaging and monitoring
ATN: Adolescent Trials Network
CARES: Comprehensive Adolescent Research and Engagement Studies
CBOs: community-based organizations
CDC: Centers for Disease Control and Prevention
CER: cost-effectiveness ratio
CLIA: Clinical Laboratory Improvement Amendments
EBIs: evidence-based behavioral interventions
HIPAA: Health Insurance Portability and Accountability Act
IRB: institutional review board
LGBTQ: Lesbian, Gay, Bisexual, Transgender, and Queer
MLMs: multilevel models
MSM: men who have sex with men
NICHD: National Institute of Child Health and Human Development
PCR: polymerase chain reaction
PEP: postexposure prophylaxis
PrEP: preexposure prophylaxis
RI: random intercept
RIAS: random intercept and slope
RCT: randomized controlled trial
SMS: short messaging service
STI: sexually transmitted infection
YABs: youth advisory boards
YAHR: youth at high risk
